# Comparing physiological responses during cognitive tests in virtual environments vs. in identical real-world environments

**DOI:** 10.1038/s41598-021-89297-y

**Published:** 2021-05-13

**Authors:** Saleh Kalantari, James D. Rounds, Julia Kan, Vidushi Tripathi, Jesus G. Cruz-Garza

**Affiliations:** Department of Design and Environmental Analysis, College of Human Ecology, Cornell University, Ithaca, USA

**Keywords:** Psychology and behaviour, Cognitive neuroscience, Perception, Short-term memory

## Abstract

Immersive virtual environments (VEs) are increasingly used to evaluate human responses to design variables. VEs provide a tremendous capacity to isolate and readily adjust specific features of an architectural or product design. They also allow researchers to safely and effectively measure performance factors and physiological responses. However, the success of this form of design-testing depends on the generalizability of response measurements between VEs and real-world contexts. At the current time, there is very limited research evaluating the consistency of human response data across identical real and virtual environments. Rendering tools were used to precisely replicate a real-world classroom in virtual space. Participants were recruited and asked to complete a series of cognitive tests in the real classroom and in the virtual classroom. Physiological data were collected during these tests, including electroencephalography (EEG), electrocardiography (ECG), electrooculography (EOG), galvanic skin response (GSR), and head acceleration. Participants’ accuracy on the cognitive tests did not significantly differ between the real classroom and the identical VE. However, the participants answered the tests more rapidly in the VE. No significant differences were found in eye blink rate and heart rate between the real and VR settings. Head acceleration and GSR variance were lower in the VE setting. Overall, EEG frequency band-power was not significantly altered between the real-world classroom and the VE. Analysis of EEG event-related potentials likewise indicated strong similarity between the real-world classroom and the VE, with a single exception related to executive functioning in a color-mismatch task.

## Introduction

Virtual environments (VEs) have been widely used by designers and researchers over the past three decades as a tool for testing products and studying human behaviors. The increasing realism of these environments provides a safe and immersive testing site in which specific design variables can be easily isolated and adjusted^1–5^. In addition to the value of precise environmental manipulation for creating rigorous study protocols, the use of VEs as a research tool can help to achieve larger study population sizes^6,7^, and can have significant cost benefits in comparison to physical prototyping^8–12^. The safety benefits of VEs compared to real-world environments can promote the inclusion of populations that might not be able to participate in real-world studies, such as elderly individuals or those with cognitive impairment^13^.

The use of VEs as a research tool supports an overall trajectory in the field toward rigorous evidence-based design. However, for this approach to be effective, it must produce ecologically valid findings that are transferable to real-world settings. One of the most significant concerns in regard to VE studies is the extent to which human behaviors, physiological responses, and psychological states seen in virtual contexts are comparable to those in natural settings.

There is evidence suggesting that particular forms of behavior and some specific physiological measurements (especially those related to stress) may be similar during virtual vs. real-world experiences^7,14–19^. In one influential study, researchers evaluated the conduct of everyday office-related activities in a physical environment vs. a closely similar VE and found no significant differences in task performance^7^. In another experiment, users’ perceptions of a real daylight environment and its equivalent representation in VR were evaluated by a self-report questionnaire, and no significant differences were found between the responses to real-world vs. virtual presentations of these environments^20^. The sense of presence in virtual environments has also been extensively studied, showing that a strong body-transfer illusion can be reliably produced by currently available VE technologies^21–25^.

Despite the promising research outlined above, the extent of this evidence is extremely limited. More benchmarking work is needed to compare, in particular, different types of physiological responses in virtual environments vs. analogous real-world conditions^26,27^. Previous studies have revealed some notable aspects of perception and experience that may affect how participants respond within VEs. For example, significant graphical distortions have been shown to pass entirely unnoticed by VE users, which may indicate that perceptual biases and expectations play a stronger role in virtual experiences compared to the real world^28^. Additionally, researchers have shown that participants who occupy a fixed position or follow a pre-determined path in virtual spaces are likely to struggle with accurate depth perception and shape perception in regard to their virtual surroundings^29–32^. In regards to learning performance, evidence has shown that VEs created a strong sense of presence, but learning performance decreased and mental working load increased compared with a 2D computer-based simulation ^33^. These studies should provide a strong caution against overly enthusiastic assumptions of generalizability from VE settings to natural ones.

**Figure 1:**
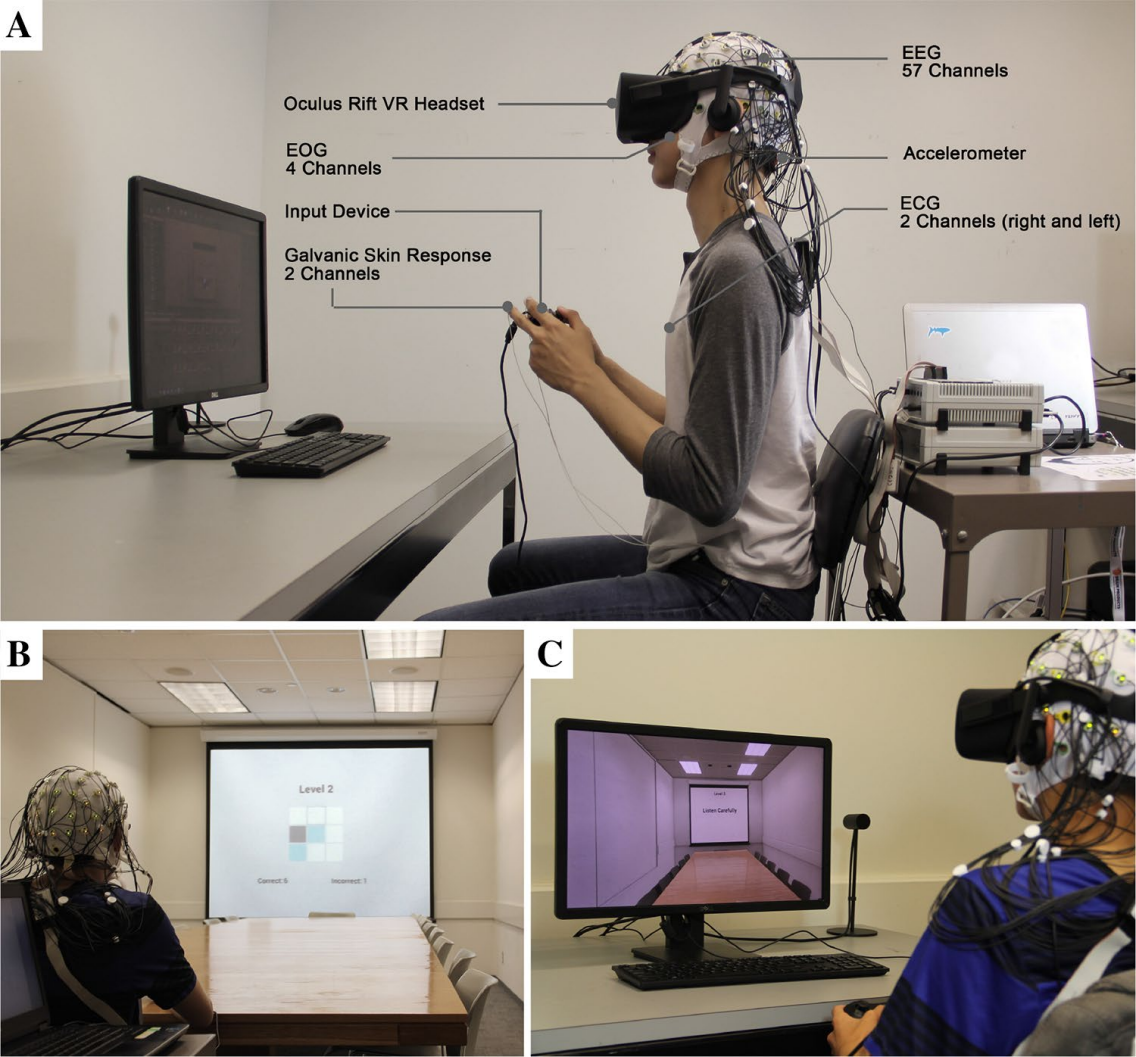
(**A**) Virtual-reality headset along with EEG, EOG, EKG, and GSR sensors, as worn by a study participant. The participants engaged in a series of cognitive tasks in (**B**)**.** the real classroom and in (**C**)**.** an identical virtual classroom. The image on the computer screen in (**D**)**.** shows a two-dimensional capture from the actual immersive 3D experience that is conveyed through the headset.

To the best of our knowledge, no prior experiment has simultaneously evaluated the physiological and behavioral performance of a set of participants using active cognitive tests in identically constructed real and virtual environments. The current study was conducted to help fill this gap, by comparing multimodal response data collected in a real classroom setting against equivalent data collected in a 3D-scanned immersive virtual representation of the same classroom (Fig. 1). The participants were asked to complete standard memory and attention tests in these environments, including the Benton Test, Visual Pattern Test, Stroop Test, Digit Span Test, and arithmetic puzzles. As they were completing these tests we recorded physiological responses using electroencephalography (EEG), electrooculography (EOG), electrocardiography (ECG), and galvanic skin conductance (GSR). The EEG data allowed us to compare a variety of metrics related to electrical activity in the brain, including frequency band-power features, and latency and amplitude in time-locked event-related potentials (ERPs). The other sensors provided data about eye blink rate, heart rate, head acceleration, and changes in skin perspiration, all of which are widely used correlates of stress.

Our primary hypothesis was that the participants’ average performance on the cognitive tests, as well as their group-level physiological responses, would be consistent between the real-world classroom and the identical virtual classroom. The ERPs were expected to differ in latency and amplitude for each of the tests performed since the tests have different levels of difficulty and require different types of cognitive activity; but within each test, we did not expect the ERP waveforms to differ significantly between the real-world and identical virtual classroom.

The choice of a classroom setting for this experiment was not merely for the sake of convenience. Among all of the various settings in which VEs are currently being applied (entertainment, medical rehabilitation, manufacturing and design industries, etc.), the educational context is one of the fastest-growing sectors^34,35^. At universities and in adult training programs there is growing excitement about the potential of virtual classrooms for enabling distance learning, and as a platform to integrate multiple modes of educational technology and presentation^36–39^. The rapidly increasing trend toward virtual classrooms, made even more relevant by the global Covid-19 pandemic, raises important questions about how students experience these environments and what effect they might have on stress, cognitive performance, and learning outcomes. The current study cannot provide a definitive answer to these overarching questions, but it offers some initial rigorous comparative evidence about participants’ performance on technical cognitive tests and physiological responses in a real-world classroom vs. an identical virtual classroom.

## Results

To compare human behavioral and physiological responses during cognitive tasks completed in identical physical and virtual environments, we precisely recreated a physical classroom in high-immersive virtual reality, including the room dimensions, furniture placement, colors, and lighting. Twenty-five participants engaged in five tests of working memory and executive functioning within each of these environments. The participants rated the realism of the virtual reality experience at 6.2 ± 1.5 using a Likert Scale where 1 indicated the experience was “not realistic,” and 10 indicated that the experience was “very realistic.” Their level of comfort with the EEG and VR head mounted display was 3.8 ± 2.2, on a Likert Scale where 1 was “uncomfortable,” and 10 was “comfortable.” Their test performance and physiological responses were evaluated in the real and the immersive VR environments for each of the five cognitive tests. The results are summarized in Table 1.

**Table 1:** Summary of statistical differences between the real-world and VR environments (test performance and physiological responses for all five cognitive tests).

	Digit Span	Benton	Stroop	Visual pattern	Arithmetic
Test performance metric: accuracy					
Test performance metric: time for completion	** t(46) = 5.53 power = 0.98 ci = [77.7 22.2] d = 1.61	** t(51) = 4.78 power = 0.95 ci = [5.2 18.5] d = 1.31	**–**	** t(48) = 6.89 power = 0.99 ci = [14.5 33.0] d = 2.59	**–**
Test performance metric: number of responses	**–**	**–**	** t(50) = -2.78 power = 0.82 ci = [-12.8 -0.2] d = -0.77	* t(53) = 2.15 power = 0.80 ci = [-0.77 7.09] d = 0.58	** t(53) = -2.43 power = 0.90 ci = [-5.84 0.27] d = -0.66
Heart beats per minute					
Eye blinks per minute					
GSR standard deviation	** t(54) = 3.73 power = 0.79 ci = [0.28 1.73] d = 0.99	** t(54) = 2.71 power = 0.59 ci = [0.01 1.11] d = 0.72			
Head acceleration standard deviation	** t(54) = 3.33 power = 0.38 ci = [0.11 0.02] d = 0.89	** t(54) = 2.74 power = 0.42 ci = [0.00 0.23] d = 0.73	** t(54) = 2.87 power = 0.32 ci = [0.02 0.45] d = 0.77	** t(54) = 2.67 power = 0.25 ci = [0.00 0.40] d = 0.71	** t(53) = 3.14 power = 0.36 ci = [0.04 0.58] d = 0.85
EEG: frequency band-power FC6. 1–4 Hz F8. 1–8 Hz AF8. 1–8 Hz		**			
IC-based ERP			**		

Full statistics are reported in-text for the EEG features. The effect sizes for significant differences are reported with the t-statistic and degrees of freedom in parenthesis: a positive number indicates that the metric was higher for the real-world environment. Statistical significance as indicated using the Kruskal–Wallis test: * p < 0.05; ** p < 0.01. Confidence intervals for the corresponding significance threshold: “ci.” Statistical power: “power.” Cohen’s d: “d.” Comparison not available due to experimental design: “–.”

**Figure 2:**
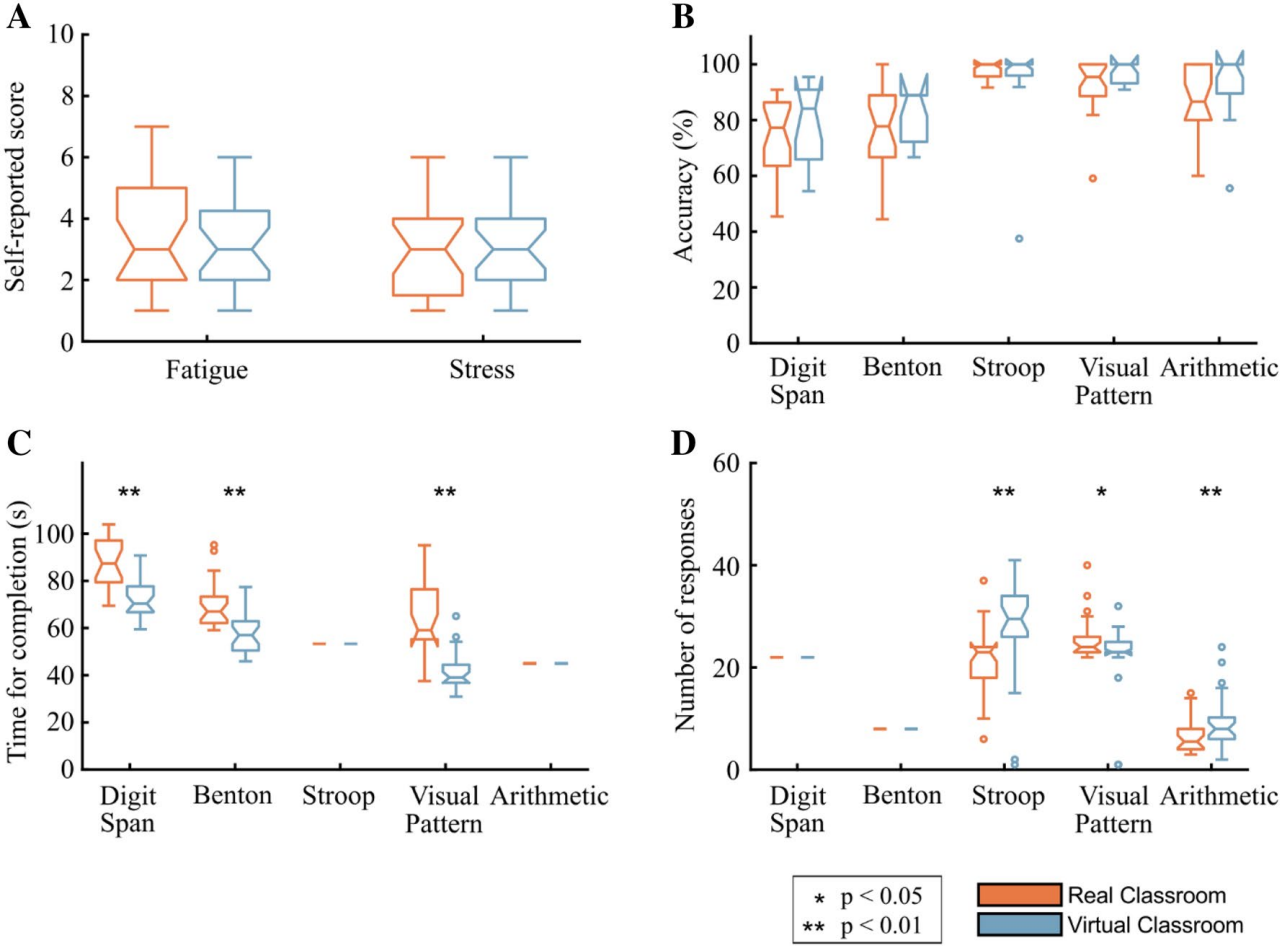
Cognitive test performance in the physical classroom environment vs. the identical virtual classroom. The color bars indicate the median scores in each environment, with the first and third quartiles shown as error bars. (**A**) Self-reported fatigue and stress levels after completing the tests, (**B**) the ratio of correct responses to total number of responses given by the participants, (**C**) the time required to complete each test, and (**D**) the total number of responses given by the participants in each tests.

The participants were asked to report their fatigue levels and stress levels after completing all five tests in each environment, using a 10-point Likert scale. No significant differences were found between the physical and virtual environments in regard to these self-reported mental fatigue [t(47) = 1.10, p = 0.27, power = 0.17, ci = [− 0.44 1.53], d = 0.37] and stress levels [t(47) = 0.36, p = 0.71, power = 0.06, ci = [− 0.74 1.06], d = 0.10] (Fig. 2A).

### Cognitive test performance

Comparing the mean accuracy scores on the cognitive tests in the physical classroom vs. the virtual classroom revealed no significant differences between these two environments for any of the five tests (Fig. 2B): Digit Span Test [t(34) = − 0.96, p = 0.34, power = 0.18, ci = [− 14.18 5.09], d = − 0.33], Benton Test [t(34) = − 0.51, p = 0.61, power = 0.07, ci = [− 11.52 6.90], d = − 0.18], Stroop Test [t(34) = 1.32, p = 0.19, power = 0.99, ci = [− 2.58 12.25], d = 0.46], Visual Pattern Test [t(34) = − 1.53, p = 0.13, power = 0.27, ci = [− 10.13 1.41], d = − 0.54], Arithmetic Test [t(34) = − 1.47, p = 0.15, power = 0.36, ci = [− 15.25 2.42], d = − 0.52]. This indicates that the participants scored equally well on the tests regardless of whether they were taken in the real-world environment or in the VE.

However, significant effects were found for the amount of time required to complete the tests. The Digit Span Test [t(46) = 5.53, p < 0.001, power = 0.98, ci = [77.7 22.2], d = 1.61], Benton Test [t(51) = 4.78, p < 0.001, power = 0.95, ci = [5.2 18.5], d = 1.31], and Visual Pattern Test [t(48) = 6.89.10, p < 0.001, power = 0.99, ci = [14.5 33.0], d = 2.59] were completed significantly faster in the virtual environment (Fig. 2C). The Stroop Test and Arithmetic Test were of fixed duration.

A significant effect was also found for the total number of responses given by the participants during the Stroop Test [t(50) = − 2.78, p = 0.01, power = 0.82, ci = [− 12.8 − 0.2], d = − 0.77], the Arithmetic Test [t(53) = − 2.43, p = 0.02, power = 0.90, ci = [− 5.84 0.27], d = − 0.66], and Visual Pattern Test [t(53) = 2.15, p = 0.04, power = 0.80, ci = [− 0.77 7.09], d = 0.58]. In the first two (time-limited tests), the total number of responses was significantly greater in the virtual classroom compared to the real-world classroom, indicating better performance. The Visual Pattern Test showed a significant decrease in number of responses submitted, indicating that there were fewer errors committed (Fig. 2D). No statistical difference with high statistical power was found for the Digit Span Test [t(53) = 2.05, p = 0.05, power = 0.33, ci = [− 11.3 − 1.81], d = − 0.77] and Benton Test [t(52) = 1.08, p = 0.28, power = 0.97, ci = [− 0.25 0.83], d = 0.29].

### Physiological responses: EOG, ECG, and GSR data

**Figure 3:**
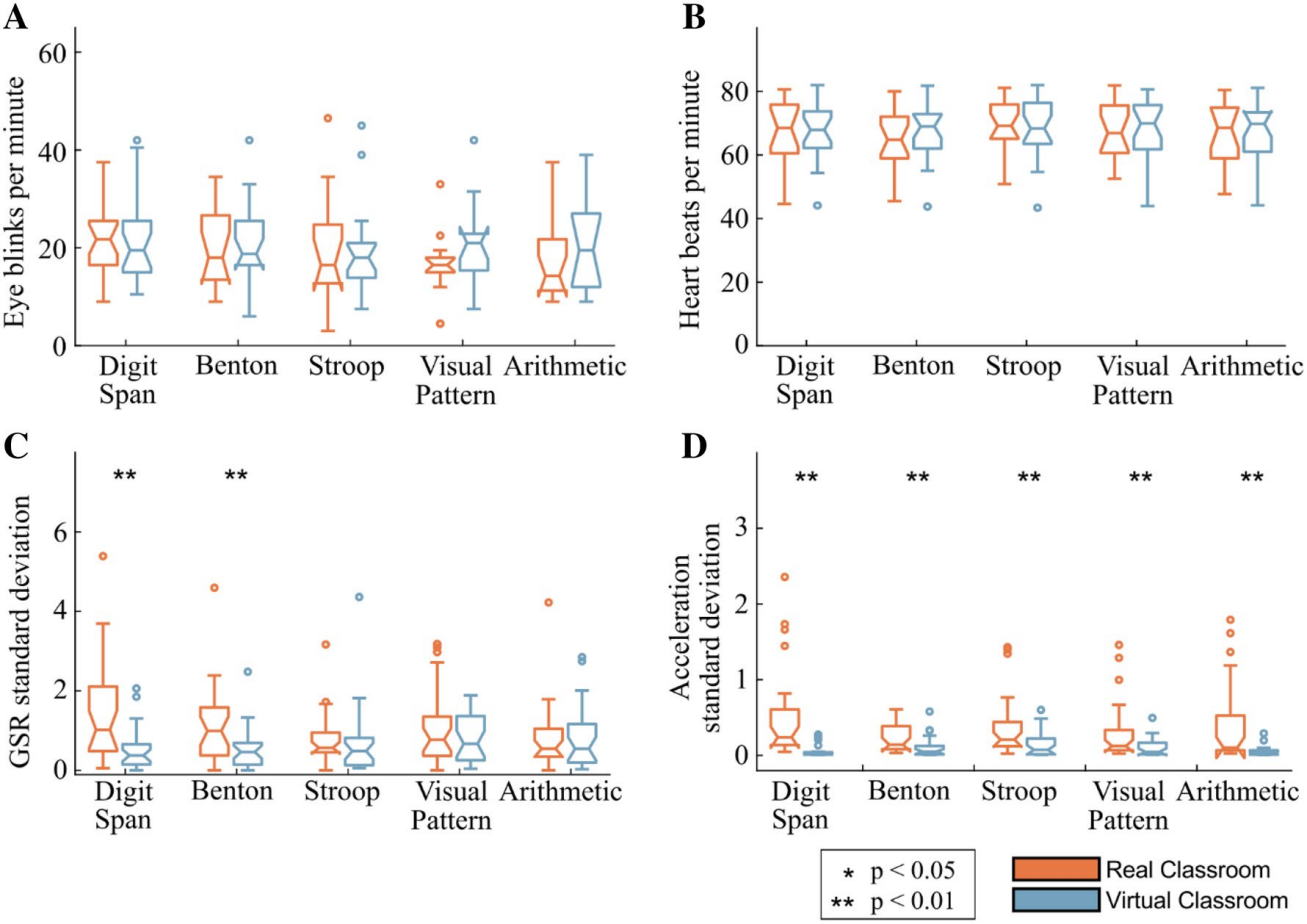
Physiological responses in the real-world classroom environment vs. identical virtual classroom. The color bars indicate the median scores in each environment, with the first and third quartiles shown as error bars. (**A**) Number of eye blinks per minute per cognitive test, as derived from the EOG sensors, (**B**) number of heart beats per minute, as derived from the ECG sensors, (**C**) relative standard deviation of tonic GSR data, and (**D**) relative standard deviation of the head acceleration magnitude.

The analysis of the vertical eye-movement (EOGv) data revealed no significant differences in the average number of eye blinks per minute in the physical classroom vs. the virtual classroom, for any of the five cognitive tests (Fig. 3A): Digit Span Test [t(42) = − 0.22, p = 0.83, power = 0.06, ci = [− 5.55 4.46], d = -0.07], Benton Test [t(25) = − 0.34, p = 0.73, power = 0.07, ci = [− 6.76 4.81], d = − 0.12], Stroop Test [t(26) = − 0.15, p = 0.88, power = 0.05, ci = [− 8.24 7.13], d = − 0.05], Visual Pattern Test [t(33) = − 2.32, p = 0.20, power = 0.031, ci = [− 10.35 2.27], d = − 0.51], Arithmetic Test [t(30) = − 0.87, p = 0.39, power = 0.015, ci = [− 9.53 3.84], d = − 0.33].

The ECG analysis revealed no significant differences in the average number of heart beats per minute in the physical classroom vs. the virtual classroom, for any of the five cognitive tests (Fig. 3B): Digit Span Test [t(19) = − 0.79, p = 0.44, power = 0.12, ci = [− 6.59 6.28], d = − 0.02], Benton Test [t(21) = 0.02, p = 0.98, power = 0.05, ci = [− 7.68 3.73], d = − 0.22], Stroop Test [t(18) = 0.51, p = 0.61, power = 0.09, ci = [− 4.04 6.92], d = 0.16], Visual Pattern Test [t(18) = 0.40, p = 0.70, power = 0.07, ci = [− 7.33 4.35], d = − 0.16], Arithmetic Test [t(14) = 0.52, p = 0.61, power = 0.09, ci = [− 6.39 5.71], d = − 0.04].

In regard to the GSR data, there was a statistically significant decrease in the variability of the per-participant standardized GSR signal in the virtual environment for the Digit Span Test, and the Benton Test (Fig. 3C). For the Stroop Test, Visual Pattern Test, and the Arithmetic Test no significant difference in GSR variability between the physical and virtual environments: Digit Span Test [t(54) = 3.73, p < 0.001, power = 0.79, ci = [0.28 1.73], d = 0.99], Benton Test [t(54) = 2.71, p = 0.01, power = 0.59, ci = [0.01 1.11], d = 0.72], Stroop Test [t(54) = 0.32, p = 0.75, power = 0.07, ci = [− 0.35 0.49], d = 0.08], Visual Pattern Test [t(54) = 1.22, p = 0.23, power = 0.17, ci = [− 0.16 0.68], d = 0.33], Arithmetic Test [t(53) = − 0.17, p = 0.87, power = 0.05, ci = [− 0.47 0.40], d = − 0.05].

Head acceleration showed a marked decrease in magnitude in the VR environments, among all cognitive tests (Fig. 3D): Digit Span Test [t(54) = 3.33, p = 0.002, power = 0.38, ci = [0.11 0.02], d = 0.89], Benton Test [t(54) = 2.74, p < 0.01, power = 0.42, ci = [0.00 0.23], d = 0.73], Stroop Test [t(54) = 2.87, p = 0.01, power = 0.32, ci = [0.02 0.45], d = 0.77], Visual Pattern Test [t(54) = 2.67, p < 0.01, power = 0.25, ci = [0.00 0.40], d = 0.71], Arithmetic Test [t(53) = 3.14, p < 0.003, power = 0.36, ci = [0.04 0.58], d = 0.85].

### Physiological responses: EEG band-power features

The EEG frequency band-power was compared between the physical and virtual classrooms, within each cognitive test. A statistically significant effect (p < 0.01) was observed in three frontal electrodes (F8, AF8, FC6) exclusively in the Delta (1–4 Hz) and Theta (4–8 Hz) bands during the Benton Test. Electrodes F8, AF8, and FC6 were found to have a significant measured increase in power in the Delta band [respectively, t(48) = − 3.45, power = 0.84, ci = [− 5.10 − 0.64], d = − 0.98; t(46) = − 2.88, power = 0.53, ci = [− 5.97 − 0.21], d = − 0.83; t(48) = − 2.54, power = 0.55, ci = [− 4.25 0.12], d = − 0.72]. In the Theta band, an increase in power was observed for electrodes F8, and AF8 in the VR environment compared to the real-world environment [respectively, t(48) = − 3.33, power = 0.81, ci = [− 3.98 − 0.43], d = − 0.94; t(46) = − 2.89, power = 0.56, ci = [− 4.43 − 0.16], d = − 0.83]. All other electrodes were not found to measure a difference in power for the five frequency bands analyzed among any of the cognitive test performed.

The F8, AF8, and FC6 electrodes measured an increase between 15 and 80 µV^2^, corresponding to 100–200% change, in mean band-power in the virtual setting compared to the real-world classroom. The differences in band-power were observed only in the lower frequency bands (1–8 Hz) in frontal electrodes. These differences are not found across the cognitive tests within these same electrodes. A Bonferroni correction for multiple comparisons (p < 7E-6) showed no significant differences in EEG frequency band-power features.

### Physiological responses: EEG Event-related potentials (ERPs)

**Figure 4:**
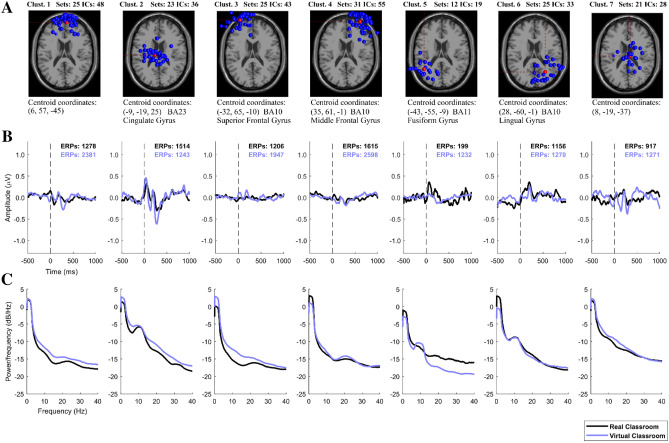
EEG group data analysis for equivalent dipoles corresponding to the ICs from all participants, in the real-world environment vs. the virtual environment. Seven IC clusters were obtained, indicated as Clust. 1–7, with their data shown in each column. (**A**) Projected equivalent dipole locations. (**B**) ERP waveforms obtained from the corresponding correct-response inputs. (**C**) ERP Power spectral density, averaged for each condition.

ERPs were analyzed to evaluate the elicited waveforms in the physical and virtual environments. The researchers analyzed the ERPs corresponding to spatial clusters of equivalent dipoles projected from Independent Component Analysis (ICA). Seven such clusters were identified (Fig. 4A), localized near the medial prefrontal cortex (Clust. 1), the cingulate cortex (Clust. 2), the left superior frontal gyrus (Clust. 3), the right middle frontal gyrus (Clust. 4), the left Fusiform gyrus (Clust. 5), the right Lingual gyrus (Clust. 6), and the center of the brain projection (Clust. 7).

*Visual inspection* of the ERP grand averages revealed major positive deflections at the cingulate cortex (Clust. 2), and Fusiform and Lingual gyri (Clust. 5 and Clust. 6) (Fig. 4B). The largest effect of the two environment conditions on ERP amplitude was found in Clust. 2, where the magnitude of the ERP corresponding to the immersive virtual reality setting was more pronounced in comparison to the real-world setting.

The *power spectral density*, a relative measure of the average strength of each constituent frequency within the ERP, was calculated for each frequency from 1 to 50 Hz, across the 1500 ms window for the ERPs in each of the two environments (Fig. 4C). Clust. 1, Clust. 3, and Clust. 4 showed alpha power suppression and a comparatively higher beta power for both environmental conditions. Clust. 2, Clust. 5, and Clust. 6 contain ICs with alpha power activation at the cingulate cortex and parietal regions.

A closer analysis was carried out to *characterize the ERPs in the medial cortical cluster* (Clust. 2). This cluster was chosen for detailed examination because it included a comparable number of components and trials from each of the two environmental datasets (Fig. 4B), it showed clear ERP patterns, and because the cognitive tests that the participants completed in this study have been reported to involve activation of the cingulate cortex in well-replicated findings using PET and fMRI scans^40–46^. Furthermore, previous high-density ERP studies have found independent sources in the cingulate cortex associated with error-detection and target monitoring^47^, and as a crucial component of response selection^21,47^.

**Figure 5:**
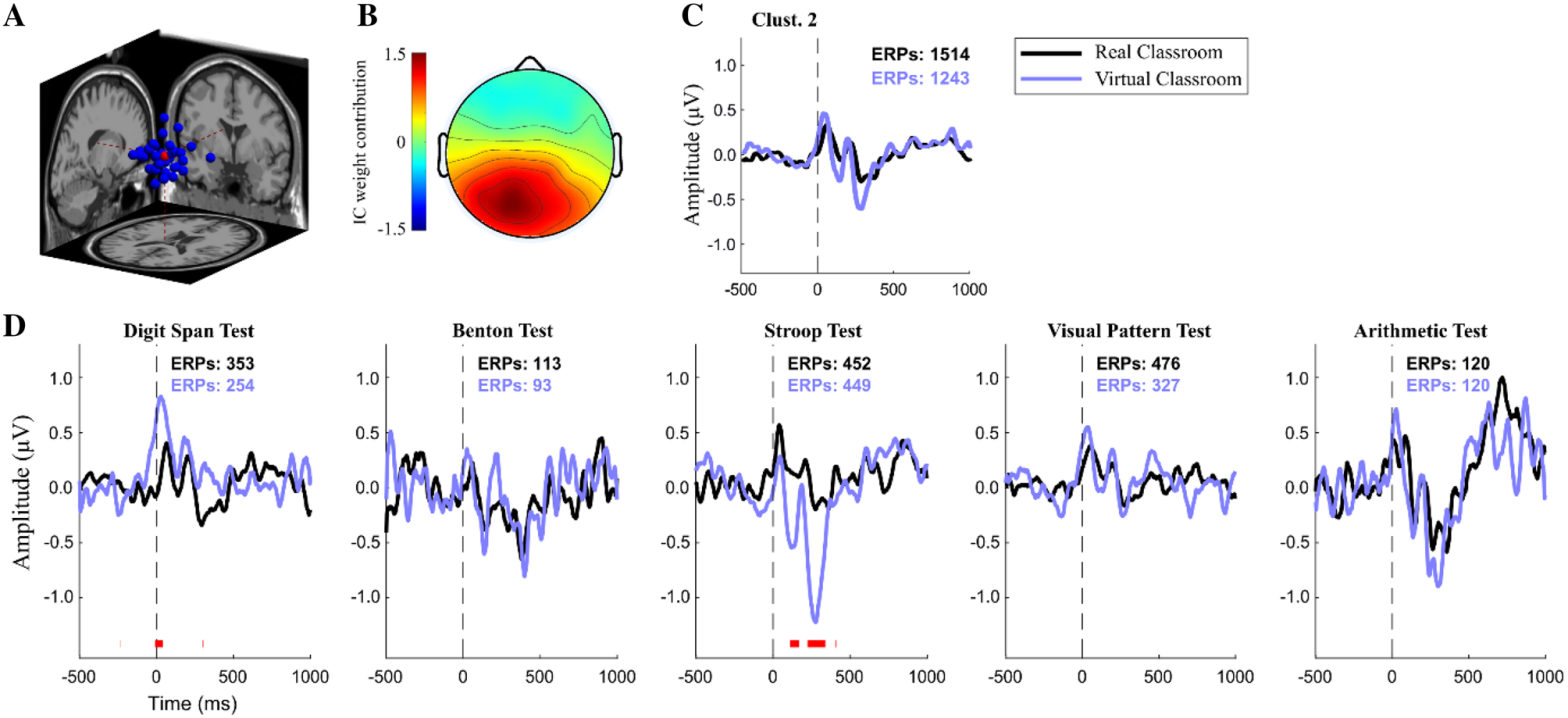
EEG data analysis, showing the ERP characterization of IC Clust. 2 across all cognitive tests. (**A**) Projected equivalent dipole location of the ICs in Clust. 2. (**B**) Scalp map representation of the averaged cluster IC. (**C**) Average waveform for all ERPs in Clust. 2. (**D**) Clust. 2 ERP waveforms for each individual cognitive test. Red marks at the bottom indicate statistically significant difference between environments (p < 0.01).

Figure 5 shows the ERPs that were obtained from the ICs located in Clust. 2 for each cognitive test, time-locked to the participants’ correct responses on the tests. The ERP patterns show specific cognitive functions recruited by the participants for each test that they performed. For the most part, these ERPs displayed very similar patterns between the real-world classroom environment and the immersive virtual environment. The overall ERP pattern found in Clust. 2 included positive peaks at 50 ms and 180–200 ms after stimulus onset, negative peaks at 130–150 ms and 270–300 ms, and then a slowly growing positive potential that peaked at 450–600 ms (Fig. 5B). The amplitude and latencies of these patterns varied in each of the cognitive tests performed: The Digit Span Test^23^ and the Benton Test^22^ showed a sustained late positive ERP component after 600 ms; while the Arithmetic Test presented a large negative potential at 270–300 ms followed by a late positive potential after 600 ms^26,48^. Significant differences in the ERP waveform between the two environments were seen during the Stroop Test, where the participants showed much greater negative peaks at 130–150 ms and 270–300 ms in the virtual environment compared to the real-world classroom (Fig. 5C). Two major ERP components have been previously described for the Stroop Test: an early effect of medial dorsal negativity around 350–500 ms, and a late effect of a positive component at 500–800 ms over the left superior temporo-parietal scalp electrodes^24^, with two dipole sources from a cluster at the cingulate cortex; consistent with the present study. Larger negativity peaks between 350 and 500 ms, as were found in the virtual classroom environment, have been previously linked to identified incongruency in the color and the meaning of the words displayed^49,50^.

## Discussion

In the COVID-19 era, many interactions and experiences that normally take place in the physical world have been ported to the digital realm, and thus understanding the psychological effects of simulated environments is more important than ever. Beyond the current widespread use of digitally mediated meetings and instruction, there is also a growing interest in using immersive virtual environments combined with rigorous physiological response-testing to evaluate the effectiveness of architectural and urban designs. This article presents behavioral, neural, and electrophysiological evidence supporting the primary hypothesis that certain types of cognitive performance mostly do not differ between a real-world classroom and an immersive virtual copy of that classroom. Specifically, cognitive test performance metrics (accuracy of completed tests), EEG ERP patterns (except for the Stroop test), EEG frequency band-power features, and several other physiological metrics did not differ significantly between the real and virtual conditions.

The overarching similarity in behavioral, neural, and physiological response data that was found between the real and virtual environments is belied in only a few measures. Statistically different effects of the virtual classroom (compared to the real-world classroom experience) included: (a) an increase in amplitude for an early 100–300 ms ERP in the medial cortical source cluster during the Stroop Test; (b) a decrease in GSR standard deviation; (c) a decrease in head acceleration; and (d) a greater response frequency on the test questions.

A notable aspect of the study design is that it used a fixed condition order, with all participants first experiencing the physical environment and then afterward experiencing the virtual environment. The reasons for using a fixed task order were logistical; this analysis was part 1 of a larger experiment block, the subsequent parts of which were all conducted using the virtual display. This made it infeasible to counterbalance the order of the conditions in the study. The use of a fixed condition order, however, raises concerns about potential habituation and trial-order effects.

Condition order effects could hypothetically cause the preponderance of null results, and an essential next step is to replicate these findings with a counterbalanced design. However, an order effect does not seem very plausible given the current data. For example, one might argue or hypothesize that entering a virtual environment should lead to decreases in frontal alpha EEG band-power (due to the increased effort needed to process the novelty of immersive media), and that a counteracting process should occur as participants become more fatigued, causing frontal alpha EEG band-power to increase with drowsiness. Since the virtual environment always followed the physical environment in this study, task-induced fatigue might counteract the stimulating effects of entering a virtual environment, leading to what seems to be a null effect. It does not seem plausible, however, that this counteracting process could cause a null effect both in the alpha band (which is inversely related to levels of neural activity), and in the beta band (which is usually positively related with levels of neural activity). Additionally, self-reported fatigue and stress did not differ significantly between the conditions.

An alternative explanation for the present findings might be that habituation/practice effects should be expected to lead to improved accuracy and decreased cognitive effort in the later (virtual) condition, but that the virtual environment also impaired cognitive ability due to increased cognitive load, leading to an overall null effect on task accuracy. This explanation is again not very plausible, because participant task accuracy was shown to suffer from a ceiling effect in both conditions, and because such practice effects on response frequency are typically observed to develop over hundreds of trials^51–53^ rather than the roughly two dozen trials that occurred in the current study. There is no evidence that a practice effect would begin to form so quickly for any of the tasks used here. In fact, practice effects in the Stroop task are not typically observed at all for response accuracy except in older adults^54^. For the Digit Span Task, a previous months-long investigation of practice effects found no significant task improvement^55^. Practice effects have been previously documented for the arithmetic task, but only over hundreds of trials^52^. These long-term practice effects do also affect neural correlates of task performance, which typically shift from greater central-executive prefrontal cortex activity during early learning to more specialized parietal cortex activity as practice leads to expertise^56^. Again, however, to the best of the researchers’ knowledge there is no prior evidence for these effects emerging over the limited number of trials that was used in the current experiment.

The participants may have visually explored the virtual classroom less because they were already familiar with its contours and features after spending time in the identical real-world classroom. This could potentially explain the lower number of head movements that were observed in the virtual environment^57^. The added weight and a possible sensation of an impeded range of motion due to the VR headset cables may have also contributed to decreased head-movements in the virtual condition, although some researchers have found that using immersive HMDs can actually increase visual search compared to a static display^58^. Other work has found that wearing mock HMDs that replicate their field of view and weight (but do not produce a virtual visual display) does not fully account for perceptual distortions common to user experience in VR, such as the distance distortion effect^59^. It cannot yet be concluded that simply wearing an HMD has a meaningful impact on perception and behavior.

The lower GSR variability in the virtual classroom may indicate less experiment-induced arousal, which typically decreases as habituation to an environment increases^60,61^. However, there is some prior evidence that the first-person immersion in virtual environments can reduce GSR-based correlates of negative affect^62^ and stress levels^62,63^.Given the short duration of the tasks, and the lack of a difference in self-reported stress or fatigue, the GSR data do not support the claim that the environments lead to different affective states. Future extensions of this research that are able to randomize the order of the experimental conditions will be able to produce more definitive conclusions about these behavioral and physiological differences that were noted in some of the experimental measures.

The greater post-response ERP in the medial cortical cluster during the virtual classroom Stroop task is less likely to be the result of the habituation phenomenon, since it only occurred with a single task and was not seen in the other classroom activities. The condition difference in GSR variability was not observed in the Stroop task, which suggests that this task may have been uniquely demanding for participants. However, mean accuracy in the Stroop task was perhaps highest of all tasks; so if the task difficulty led to SNS arousal, participants were able to self-regulate and meet the challenge. In our implementation of the Stroop Test, the words were presented using a projector and screen in the real-world classroom, while in the virtual classroom these words were displayed as part of the VR interface. It is possible that the differences observed in the amplitude of negative components in the ERPs could be a consequence of variations in the sharpness of the color displayed in each setting. This could indicate that visual processing of colors during the Stroop Test was more effective in the virtual environment compared to the real-world classroom due to higher color legibility^64^. However, this does not explain why the GSR levels were relatively higher in the VR Stroop test compared to the other VR tests.

No significant differences were found in the EEG frequency band-power data between the real-world classroom and the virtual classroom when using Bonferroni correction for multiple comparisons. However, before this family-wise error correction, occasional differences between conditions were found in frontal electrodes in the Delta and Theta bands (1–8 Hz), for F8, AF4, and FC6. The location of these electrodes suggests that the differences could have been due to pressure applied by the VR headset as it rested on the EEG cap. However, these differences were observed almost exclusively for the Benton Test, making it unlikely that the HMD caused an artifact that affected all EEG data in the virtual condition. Our ERP results did not suggest condition differences associated with the Benton test. Furthermore, given that the statistical differences at p < 0.01 were found for less than 0.4% of all EEG band-power comparisons, the results suggest that these EEG features remained mostly unaffected by the transition to a virtual environment.

The findings of the current study conflict with a few results that have been produced by other researchers. In a recent PC-based STEM learning task study by Makransky and colleagues^33^, EEG correlates of cognitive workload were found to be higher in the immersive VR version of the task, while learning performance was lower than in a non-immersive version^33^. Nevertheless, the participants in that study reported enjoyed the immersive version more than the PC-based version. Whether this hedonic quality is a function of the novelty of the VR experience, the different sense of presence compared with normal waking life, or some other set of qualities remains to be determined. In other work by this group, using different tasks, virtual/immersive-based training actually led to improved learning performance compared to in person or desktop-PC (2D) training^65^. Further work will need to be done to replicate cognitive workload results and further examine the effects of virtual environments on learning performance before reaching strong conclusions.

It should also be noted that the current study examined only a small range of learning-related activities, specifically cognitive tests of visual memory, concentration, and mathematical reasoning. In other research, participants have been asked to interact with and manipulate virtual “objects,” which may lead to entirely different forms of physiological response, subjective feelings, and learning outcomes^66,67^. Other work has focused on the effects of immersive environments on sense of presence and embodiment, when a visualized avatar is part of the experience^68,69^. However, the current work offers a simple baseline comparison of virtual and physical environments, with no manipulations of embodiment or object interaction. Further work should measure these activities, and many other forms of learning and engagement, while comparing physical environments to visually identical virtual environments. These findings provide evidence for neural-based features that can be reliably generalized from virtual spaces to real ones. In the educational context, further longitudinal studies are needed to evaluate long-term learning experiences and outcomes in physical vs. virtual environments.

The results of the current study are contingent upon the particular type of environment that was examined (a small windowless classroom), the profile of the study participants, and the types of cognitive tests performed. Nonetheless, the data collected during the study support the conclusion that VEs have a strong potential for use as a research tool, and that many of the findings obtained in VEs may be transferrable to real-world settings. The study also tentatively supports the conclusion that virtual classrooms can allow for similar student comfort and outcomes compared to real-world classrooms, at least in the context of short-term measurements of fatigue, stress, and cognitive performance on technical tests. By further understanding the transferability of various types of human response data between virtual environments and real ones, researchers will be able to establish standardized VE testing as a source of “big data” for the design industry, with behavioral and physiological measurement capabilities integrated into an inexpensive and effective research platform.

## Materials and methods

The study protocol was reviewed and approved by the Institutional Review Board at the University of Houston, and all participants provided written informed consent to participate in the study. Informed consent for publication of identifying images in an online publication was obtained for the study. All experiments were performed in accordance with relevant guidelines and regulations.

In the current experiment, we scanned the texture of the materials in the real classroom, then imported this data into the Unreal Engine (www.epicgames.com) to create a high-dynamic-range (HDR) immersive virtual rendering. The detailed 3D scan allowed for the real classroom’s spatial dimensions, colors, lighting, furniture placement, and all other features to be precisely replicated in the VE. The researchers created a widget that would allow the cognitive tests to be displayed on a projector screen, which was included both in the real classroom and in the virtual classroom (Fig. 1B,C).

The virtual experience was presented to the research participants using an Oculus Rift head-mounted display, which uses a 1920 × 1080 pixel low persistence organic light-emitting diode display with a refresh rate up to 75 Hz, resulting in a resolution of 960 × 1080 pixels per eye. The headsets are lightweight, comfortable for the user, and have a strong market share with ongoing development. The participants interacted with the VE using a handheld controller: a joystick was used to toggle between multiple-choice answers for the cognitive tests, and a button-press indicated the selection of the answer (Fig. 1A).

We selected physiological sensors and associated software that could be easily integrated and that would allow for rigorous data collection. An important consideration was that the sensor components should allow a wide range of head motion, as is necessary for VR-headset use, while still providing robust data outputs. The selected unit was a 63-channel actiCHamp (Brain Products GmbH). 57 electrodes were used for EEG recordings, 4 electrodes were used to collect EOG data (two placed laterally 1 cm outside the eyes, and two vertically from the right eye), and the final two were used for ECG measurement. A GSR module (Brain Products GmbH) was used to record skin conductance (Fig. 1A), and a tria-axial accelerometer was used to collect head acceleration. All physiological and acceleration metrics were synchronized through the actiCHamp, and the participant responses were synchronized with the rest of the data through Lab Streaming Layer^70^. The BrainVision Recorder software package was used for all electro-physiological data-recording, and EEGLAB^71^ software was used for data cleaning and analysis.

### Participants

An a-priori analysis of the required sample size was conducted using G*Power (Version 3.1.9.3.). For an effect size of dz = 0.55, a 0.05 probability of error, and a power of 0.80, the necessary sample size was 27. The effect size of dz = 0.55 was estimated from Makranksky and colleagues'^33^ effect size for frequency band-power based cognitive load change comparing a PC display vs. immersive VR. Technical recording issues discovered early in the data-collection process led to four participants being excluded from the study, but the researchers were able to recruit replacements to reach the intended sample size of 27. During the subsequent EEG data-analysis two additional participants were discovered to have severe data quality issues that also necessitated exclusion, resulting in a final sample size of 25.

The ages of the 25 participants ranged from 18 to 55 years (M = 25.67, SD = 7.47). Fourteen reported as male and 11 reported as female. All participants had normal or corrected-to-normal vision. Each participant gave informed written consent before participating in the experiment, and was compensated with a $25 gift card after completing the tasks.

### Research protocol

The research protocol consisted of four stages. In Stage 1, each participant was provided with an introduction to the study and its goals, asked to sign a consent form and complete the demographic questionnaire, was fitted with the relevant sensor devices and allowed to “play” with the virtual headset, and then sat still for a short time without the VR headset so that resting-state EEG data could be collected: one minute of rest with eyes open and one minute of rest with eyes closed. In Stage 2 the participants completed cognitive tests in the physical classroom environment (without using the VR headset), and then rated their level of fatigue on a Likert scale from 1 to 10. In Stage 3 they donned the VR headset and completed the same cognitive tests in the virtual classroom environment, and then again rated their level of fatigue. Finally, in Stage 4 the sensor equipment was removed and the participants completed an exit interview. Participants were asked at this time to provide feedback about their overall experience in the VR environment, and they used Likert Scales to rate the realism of the VE (from 1 = “not realistic” to 10 = “very realistic”) and the comfortableness of the EEG headset (from 1 = ”uncomfortable” to 10 = ”comfortable”).

### Cognitive tests

The cognitive activities used in the study included five standard memory and attention tests.

The *Digit Span Test* is part of the Weschler Adult Intelligence Scale-Revised (WAIS-R)^72^. It was used to assess the participants’ working memory, attention, encoding, and auditory processing. The test consisted of the participants listening to sequences of digits and then repeating those digits back to the researcher in the correct order. The study used two sequences of four digits, one sequence of six digits, and finally one sequence of seven digits. The participants received visual and auditory feedback indicating correct or incorrect answers. There was no time limit for this test.

The *Benton Visual Retention Test*^73,74^ is a measure of visual perception, visual memory, and the capacity for visual discrimination. In this test, the participants observed a complex visual stimulus for three seconds, followed by a set of four visual options. One of the options was identical to the original stimulus, whereas the other three options had subtle differences from the original. The participants were asked to select the figure that matched the original stimulus. This test was conducted eight times, using a variety of different visual stimuli. As per the test protocol, no feedback was given about correct or incorrect answers, and there was no specific time limit.

The *Stroop Color Interference Test* is one of the most extensively studied metrics in cognitive psychology^75^. This task consisted of identifying the color of a word presented on the screen. Sometimes the word matched its color (e.g., the word “blue” presented in blue ink); whereas other words conflicted with their color (e.g., the word “blue” presented in black ink). The congruent and incongruent words were presented randomly, with a new word appearing immediately after every answer. The participants received visual and auditory feedback for correct or incorrect answers and were asked to try to get as many correct answers as possible within 45 s.

During each trial of the *Visual Pattern Test,* the participants were presented for 2 s with a grid consisting of light and dark squares^76^, which was then was replaced by a blank (all-light-colored) grid. The participants were asked to fill out the grid by selecting the correct dark squares to match the original that they had previously seen. Four iterations of this test were conducted—two iterations using a 3 × 3 grid with four randomly darkened squares, and then two more iterations using a 4 × 4 grid with seven randomly darkened squares. The participants received visual and auditory feedback for correct or incorrect answers. There was no specific time limit for this test.

The *Arithmetic Test* consisted of evaluating two simple arithmetic statements (e.g., 9 + 12 and 7 + 20). The participants had to choose which of the two statements was greater, or else select that both statements were equal. After a participant selected an answer, a new set of arithmetic statements was immediately presented. The participants received visual feedback for correct or incorrect answers, and were asked to try to get as many correct answers as possible within 45 s.

The cognitive test results were evaluated according to the relevant scoring metrics for each test. For example, scores on the Digit Span Test were recorded as the total number of correct digits out of a maximum of 22 possible inputs (4 digits + 5 digits + 6 digits + 7 digits). Although no specific time limit was given for this test, the time of completion was recorded and was used as part of the scoring matrix (as per the standard Digit Span Test protocol). The Stroop Test, in contrast, was scored according to the ratio of correct answers to the total answers submitted (again as per the standard test protocol). The participants’ scores on the cognitive tests were analyzed at the group level, by looking for significant differences between the average scores in the real classroom and the average scores in the identical virtual classroom. A Kruskal–Wallis statistical test was used with a significance level of p < 0.05 and p < 0.01.

### EEG data processing

The EEG data were analyzed using the EEGLAB software package^71^. Raw .xdf data files were imported at their original sampling rate of 500 Hz. They were low-pass filtered at 100 Hz, high-pass filtered at 0.1 Hz, and then run through the Cleanline algorithm, which selectively filters out the 60 Hz power-line noise using an adaptive frequency-domain (multi-taper) regression technique. Artifactual time-windows and channels were deleted using manual inspection, by research team members blind to the order of the trials. The data were then run through an independent component analysis (ICA) using the extended Infomax algorithm, and a dipole-fitting step using the Dipfit package, with the source-space warped to fit a template MNI space^77,78^. Artifactual components representing eye-movements, head movements, and other gross non-brain components were removed by visual identification.

One of the primary EEG metrics evaluated in the study was the *frequency band-power features*. This measurement indicates how strongly a particular frequency or frequency range contributes to an EEG time-series signal, typically over a selected time window. Frequency band-power features were computed using 40-s windows beginning at the start of each cognitive test. The Multitaper power spectral density estimate function^79^ in Matlab was used to calculate the band power, with a time-half-bandwidth product of 4. The log-transformed (dB) EEG power was analyzed for all statistical comparisons. The band power features were calculated for five typical EEG frequency bands: Delta (1–4 Hz), Theta (4–8 Hz), Alpha (8–12 Hz), Beta (12–30 Hz), and Gamma (30–40 Hz).

The frequency band-power features were analyzed at the group level by comparing the frequency band-power distribution from all participants in the VR and real conditions. For each cognitive test, the distribution of frequency band-power from all participants in the VR condition was compared to the distribution in the real classroom. A Kruskal–Wallis test was used for each EEG electrode and each frequency band, with a significance level of p < 0.01. A Bonferroni correction for multiple comparisons (5 cognitive tests, 5 frequency bands, and 57 channels), yields a statistical significance level of p < 7E-6.

The second EEG metric evaluated in source space was *event-related potentials* (ERPs). Epochs of EEG data were time-locked using button-presses whenever the participant answered, over the range of – 500 to 1000 ms after the button-press. Only correct trials were included for the analysis, and the resulting averaged cluster ERPs were then smoothed by taking a 20-point moving average. We compared cluster ERP amplitude between environment conditions as described in the Statistical Analysis section below. The ICA activations from this ERP data were taken for analysis, calculated separately for each participant and environment condition. An equivalent dipole was calculated for each IC using a boundary element head model based on the MNI brain as implemented by DIPFIT routines^80^. ICs for which the dipole location estimation explained less than 20% of the variance in that IC’s activity were excluded. By using the EEGLAB pre-clustering function, distances between all ICs were calculated with their equivalent dipole model locations, with the standard weighting of 1. The IC equivalent dipoles were clustered into seven groups using k-means as implemented in EEGLAB, which clustered components from different subjects based on Euclidean distance from within the common MNI brain structure. IC equivalent dipoles that were further than three standard deviations from a cluster centroid labeled as outliers and omitted from further analysis. Seven clusters were generated (in addition to an 8th for outliers)—this number of clusters was chosen by a manual inclusion heuristic—seven was a low enough number to maximize the number of participants who had components recruited to each cluster, while high enough to prevent distinct brain signals from being aggregated together into overall general clusters^81–83^. MNI coordinates were converted to Talairach using the Tailarach Client 2.4.3 (ICBM), which was also used to identify the Brodmann areas in which each cluster centroid was putatively located. Average ERPs were computed for each task, for each cluster, from − 500 to 1000 ms time-locked to the correct responses within each task, for each cluster, for both environments. The trial amplitudes were baselined to − 500 to 0 ms in the ERP time window. The ERP plots were compared using a two-tailed t-test between each data point to identify any significant differences between the two environments.

### EOG data processing

Ocular electrodes were placed above and below the left eye to measure the vertical EOG data, and left and right temples to measure the horizontal ocular movement. Eyeblink occurrences were identified with the BLINKER toolbox EEGLAB extension implementation^84^, with default parameters using frontal channels the upper and lower EOGv, Fp1, F1, Fp2, Fz, Fpz, F3, F4, and F2 as candidate signals. Horizontal EOG electrodes were used to record saccades and lateral eye movements but were not used in this analysis. Eye blinks are of interest in this experiment because of their prevalence as the main source of artifact contamination in EEG recordings^85^. Furthermore, as a physiological measurement in virtual and real environments, the rate of eye-blinking can be an index of several affective and cognitive states or changes, including stress, fatigue, and engagement^86^. The eye-blink data were analyzed at the group level, by using a Kruskal–Wallis test to compare the average rate of eye-blinks across participants between the two environments.

### ECG data processing

Two electrodes were placed on the chest and used as ECG sensors, following the standard locations for RA-LA in the Einthoven I derivation^87^. The purpose of collecting ECG data was to measure the heartbeats per minute. The ECG data were filtered between 4 and 10 Hz using a fourth-order Butterworth filter, and then the heartbeats per minute were calculated using the Heart Rate Variability toolbox^88,89^. The heartbeats per minute data were analyzed at the group level to identify any significant differences between the participant averages in the real classroom vs. the virtual classroom.

### GSR data processing

The GSR data was obtained with an Ag/AgCl electrode pair on the tip of the index and middle fingers from the non-dominant hand of the participants. The GSR data was de-trended by removing the best straight-line fit for each participant. Changes between the real and the virtual environments were analyzed by extracting the GSR signal from the first 40 s of each cognitive test, for each participant. The extracted data was standardized (z-score) per participant, by subtracting the mean and dividing by the standard deviation. The variability of the obtained z-scores was compared statistically (Fig. 5C) between environments.

### Head acceleration

The head acceleration data was collected from an integrated tri-axial accelerometer, synchronized with the electrophysiological data through the actiCHamp module. The acceleration magnitude was obtained by calculating the square root of the squared components. In a similar analysis as for the GSR data, changes between the real and the virtual environments were analyzed by extracting the acceleration signal from the first 40 s of each cognitive test, for each participant. The data was then standardized per participant, by subtracting the mean and dividing by the standard deviation. The variability of the obtained z-scores was compared statistically (Fig. 5D) between environments.

### Statistical analysis

The statistical significance for each comparison was obtained using the nonparametric Kruskal–Wallis test with a significance level of p < 0.05 and p < 0.01. Additionally, the statistical power for each comparison was calculated post-hoc^90^, given the number of samples, mean, and standard deviations in the distributions for a two-sampled, unpaired t-test comparison. The statistical results are given for each comparison that produced significant differences by showing the t-statistic followed by the degrees of freedom in parentheses, statistical power, confidence intervals for the significance level threshold reported, and Cohen’s d. The data distributions are shown as boxplots in Figs. 2 and 3. The 95% confidence interval endpoints are represented by a notch in the boxplots.

## Data Availability

All data and analysis scripts related to this paper will be made available in the Open Science Framework (https://osf.io/).
